# Supplementary material for: A systems-level framework for drug discovery identifies Csf1R as an anti-epileptic drug target
Source: Nat Commun. 2018 Sep 3;9:3561. doi: 10.1038/s41467-018-06008-4 (PMC6120885; doi:10.1038/s41467-018-06008-4)
Supplement: Supplementary file 1 — Supplementary Information [file 41467_2018_6008_MOESM1_ESM.pdf]

Supplementary Figure 1: Hierarchical cluster analysis.

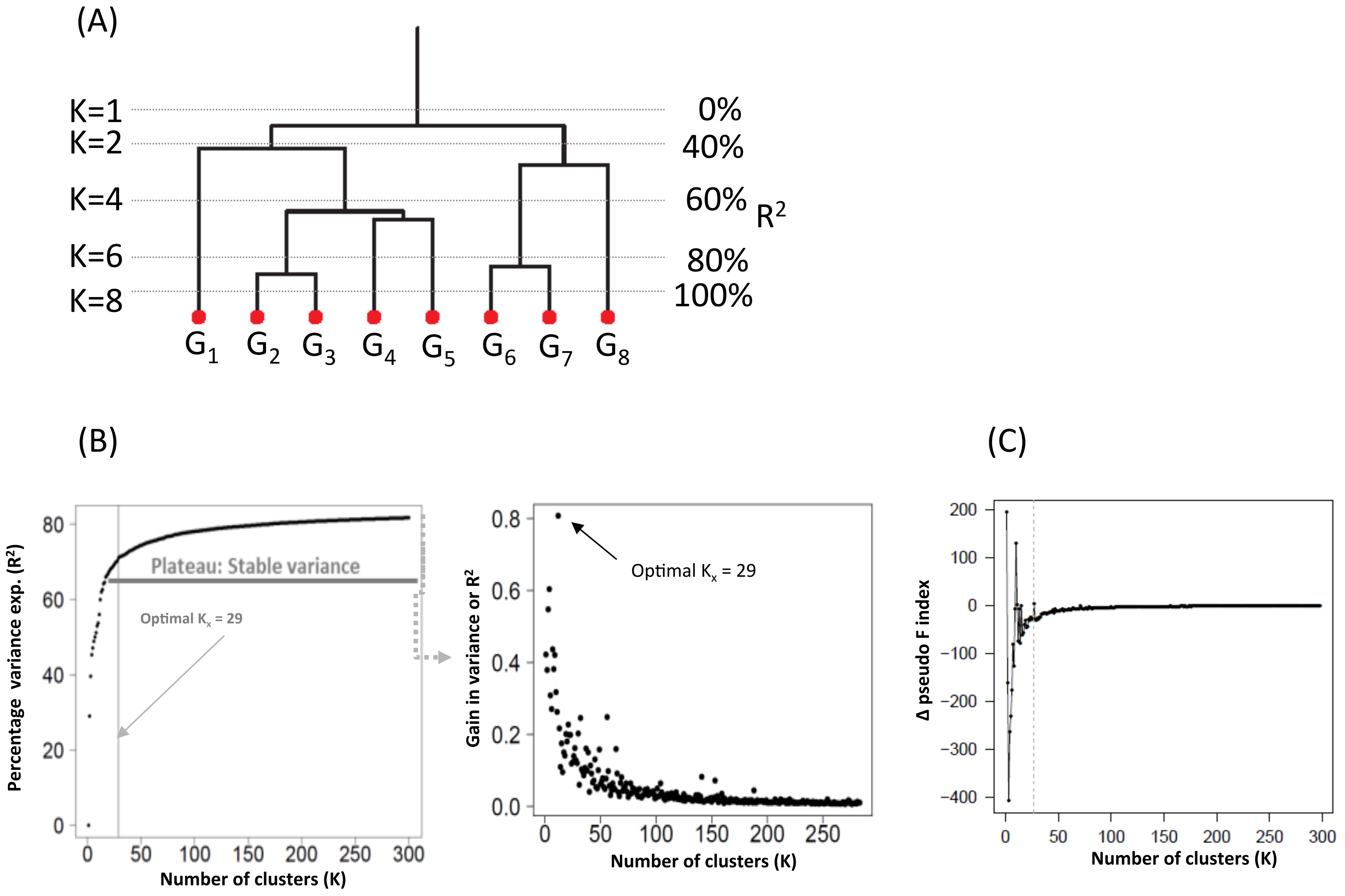

Supplementary Figure 1: Hierarchical cluster analysis. Ward's method looks at cluster analysis as an analysis of variance problem - at each step of the analysis clusters are merged so as to produce the smallest increase in the sum-of-squares error term. **(A)** We show the relationship between the number of clusters and the variance explained. For example, when the data are not clustered (K=1) then no variance is explained, whereas 100% of the variance could be explained if K= all genes in the cluster analysis. **(B)** To identify a point of stable variance (elbow), we used percentage of variance explained (R^2) and plotted it with increasing number of clusters. **(C)** The panel shows delta pseudo F index value and the dotted grey line shows the optimal number (Kx=29) of clusters before reaching a plateau of stable F index value. The Pseudo F index describes the ratio of between-cluster variance to within cluster variance and is measure of cluster compactness as well as separation.

Supplementary Figure 2a: Module gene ontology (GO) pathway enrichment.

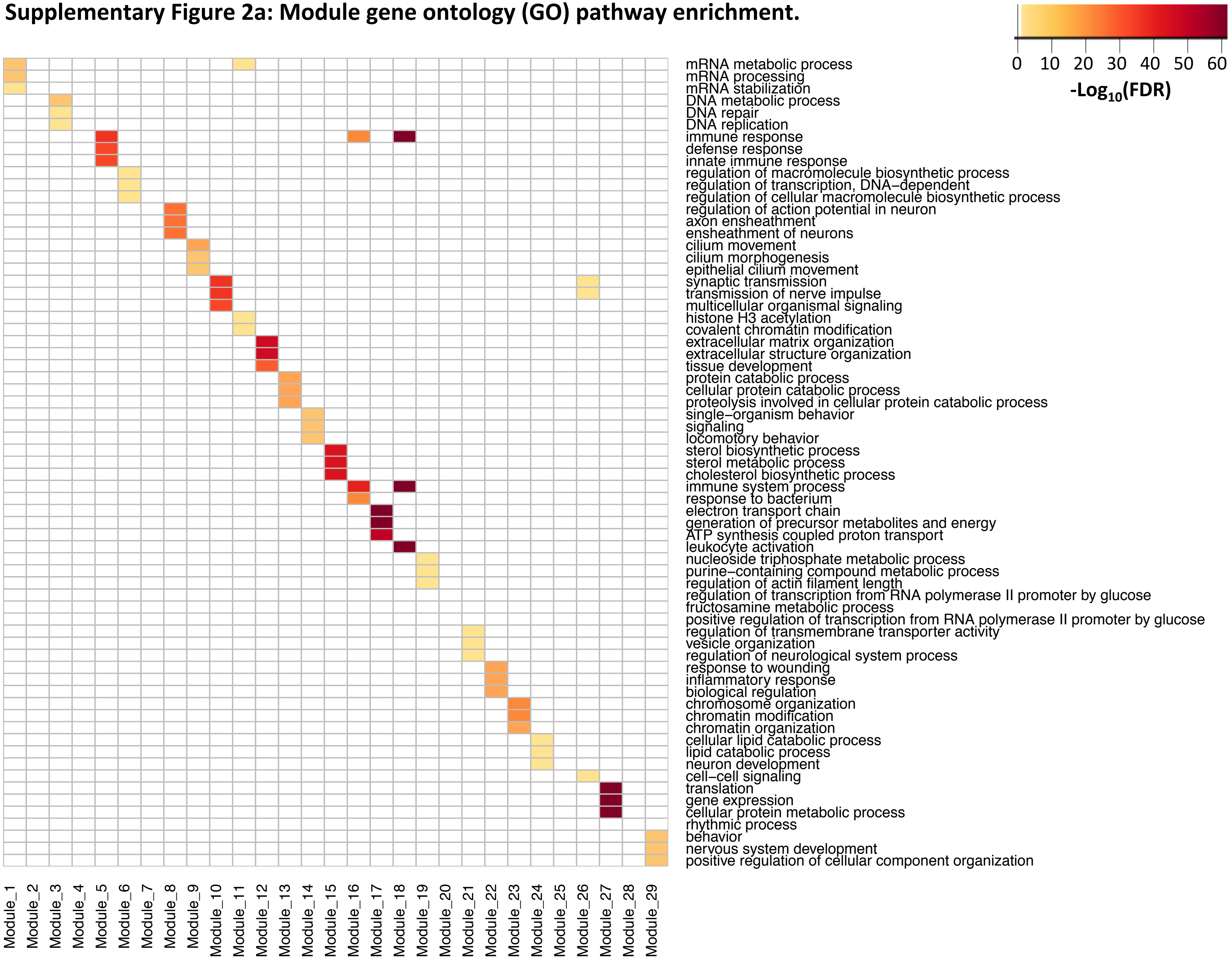

Supplementary Figure 2b: Module cell type enrichment analysis.

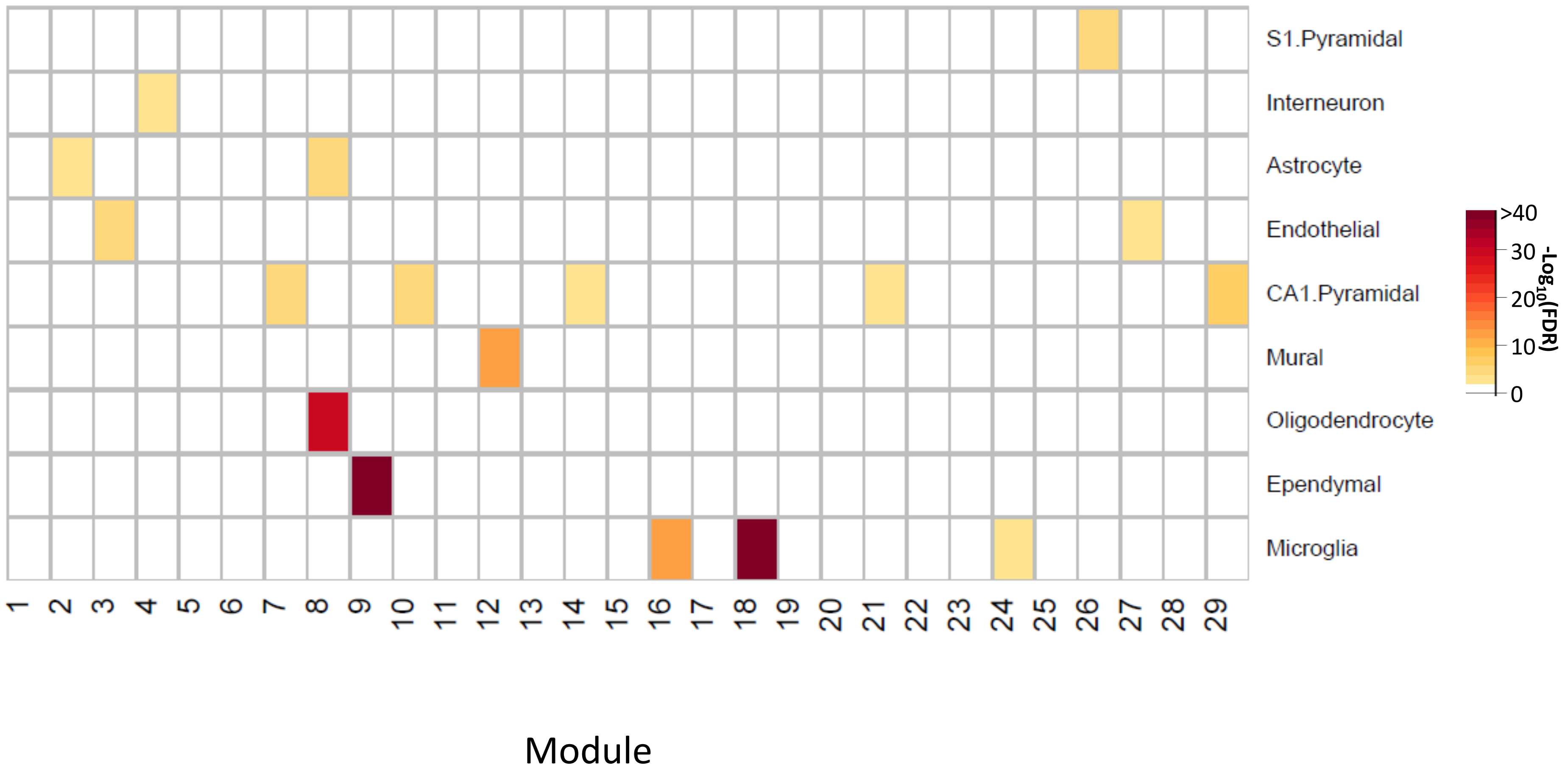

Supplementary Figure 2: (A) Module gene ontology (GO) pathway enrichment. Figure showing top three significant (FDR<0.05) Gene Ontology (GO) enrichments for each module from the epileptic mouse hippocampus. The FDR for each GO term is represented by the color scale - the darker the color the more significant the result. **(B) Module cell type enrichment analysis.** Cell type enrichments for the epileptic hippocampus co-expression modules (FDR for each cell-type enrichments represented by the color scale).

Supplementary Figure 3: Summary of module prioritization.

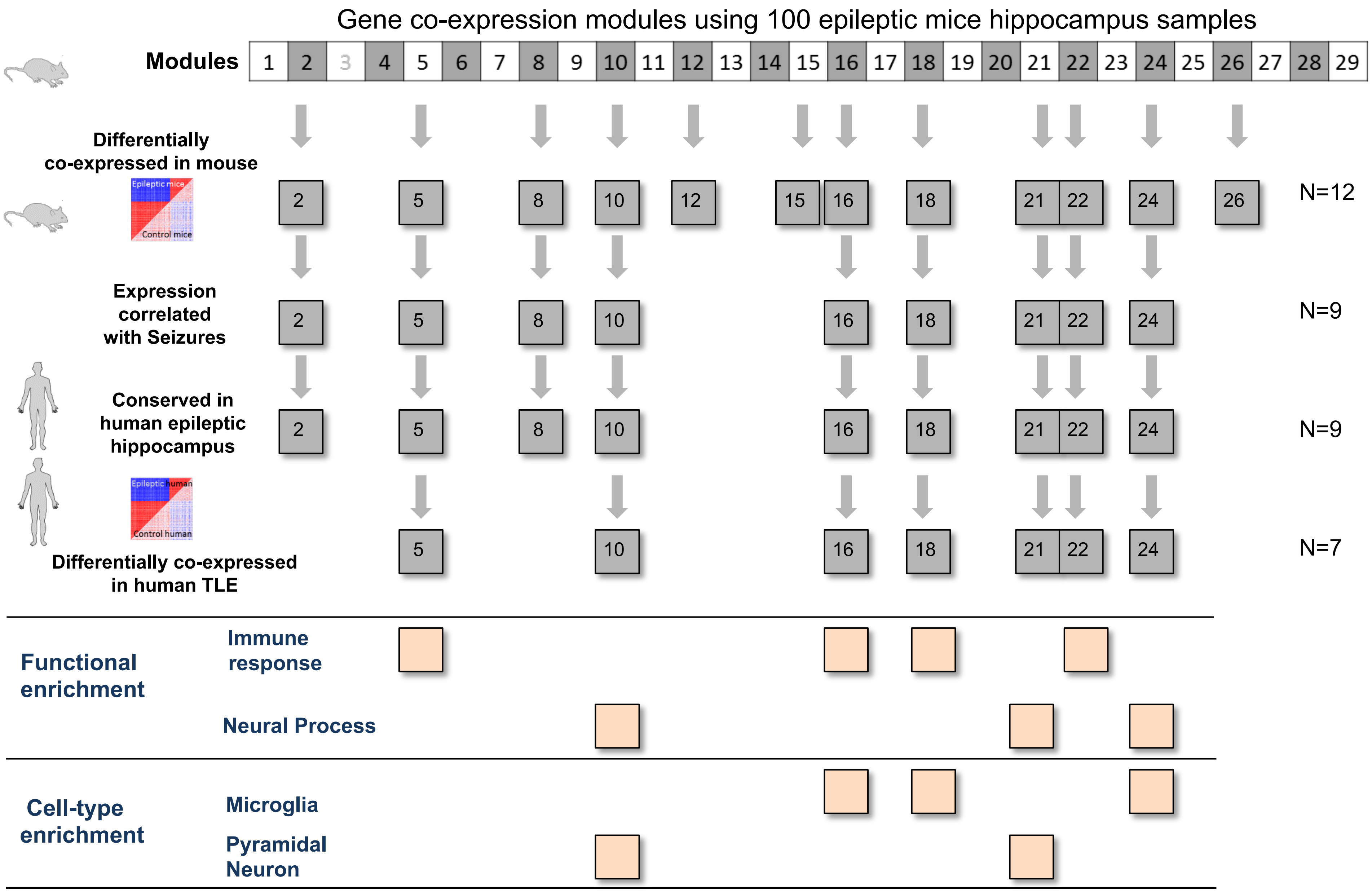

Supplementary Figure 3: Summary of module prioritization. Of the 29 modules in the mouse epileptic hippocampus, 12 were differentially co-expressed between epileptic and control mouse hippocampi. Nine of these 12 modules had a pattern of expression that correlated significantly with seizure frequency. All nine of these were conserved in the human epileptic hippocampus and the seven that were also differentially co-expressed in the human epileptic hippocampus were taken forward for CRAFT analysis. Module functional and cell type enrichments are shown, with modules 16 and 18 enriched for immune response pathways and expressed in microglia, and modules 10 and 21 enriched for neural processes and expressed in pyramidal neurons.

Supplementary Figure 4: Correlation patterns in co-expression modules.

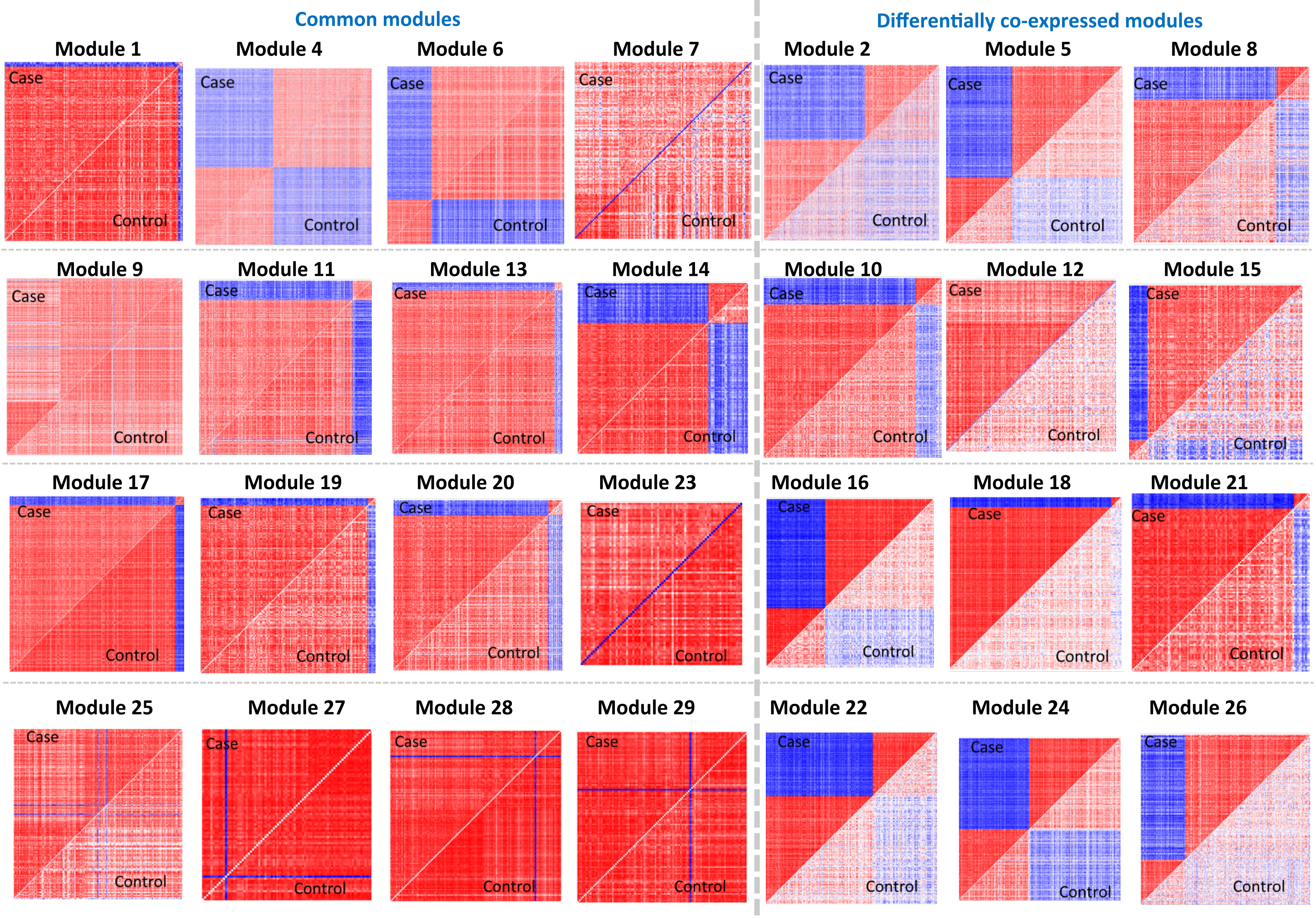

Supplementary Figure 4: Correlation patterns in co-expression modules. The figure summarizes the co-expression patterns of the modules between the hippocampus in epileptic and control mice. The thick ‘grey’ dotted line in the figure separates the set of modules that were significantly differentially co-expressed between epileptic cases and controls (right) from those whose correlational structure was similar in cases and controls (left). The Spearman’s correlation coefficients are represented in the form of a color scale with shades of blue to red representing negative and positive correlation between gene pairs within the modules.

Supplementary Figure 5: Clinical characteristics of epileptic mice.

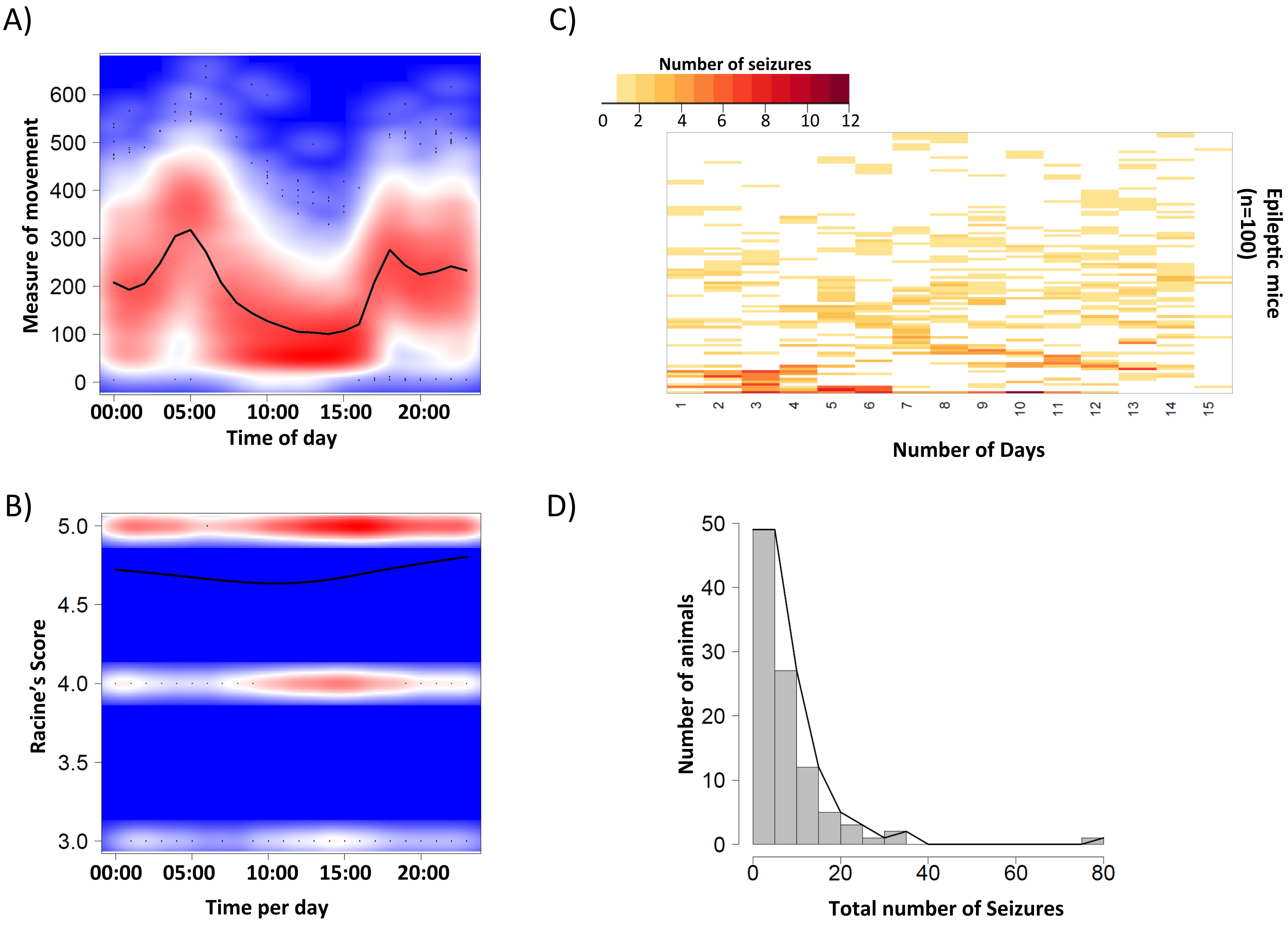

Supplementary Figure 5: Clinical characteristics of epileptic mice. Movement and seizure data for the epileptic mice cohort measured by 14 days of continuous motion sensing 3D accelerometry synchronized with continuous video monitoring starting on day 28 post pilocarpine-induced status epilepticus. The figure summarizes the different parameters of movement and seizures in epileptic mice during the monitoring period. **(A)** Density plot showing the circadian rhythm of epileptic mice. Here, the X-axis shows time of day and the Y-axis shows an arbitrary measure of mouse movement. **(B)** Density plot summarizing the intensity of seizures represented by Racine's score (Y-axis) for each seizure that was observed for the time of the day (X-axis) during the monitoring period. **(C)** Heatmap summarizing the number of seizures observed in each mouse (Y-axis) over the 14 days of video monitoring (X-axis). The number of seizures are represented in the form of color scale – the deeper the color the higher is the number of seizures. **(D)** The histogram shows the distribution seizures during the monitoring period.

Supplementary Figure 6: CRAFT workflow.

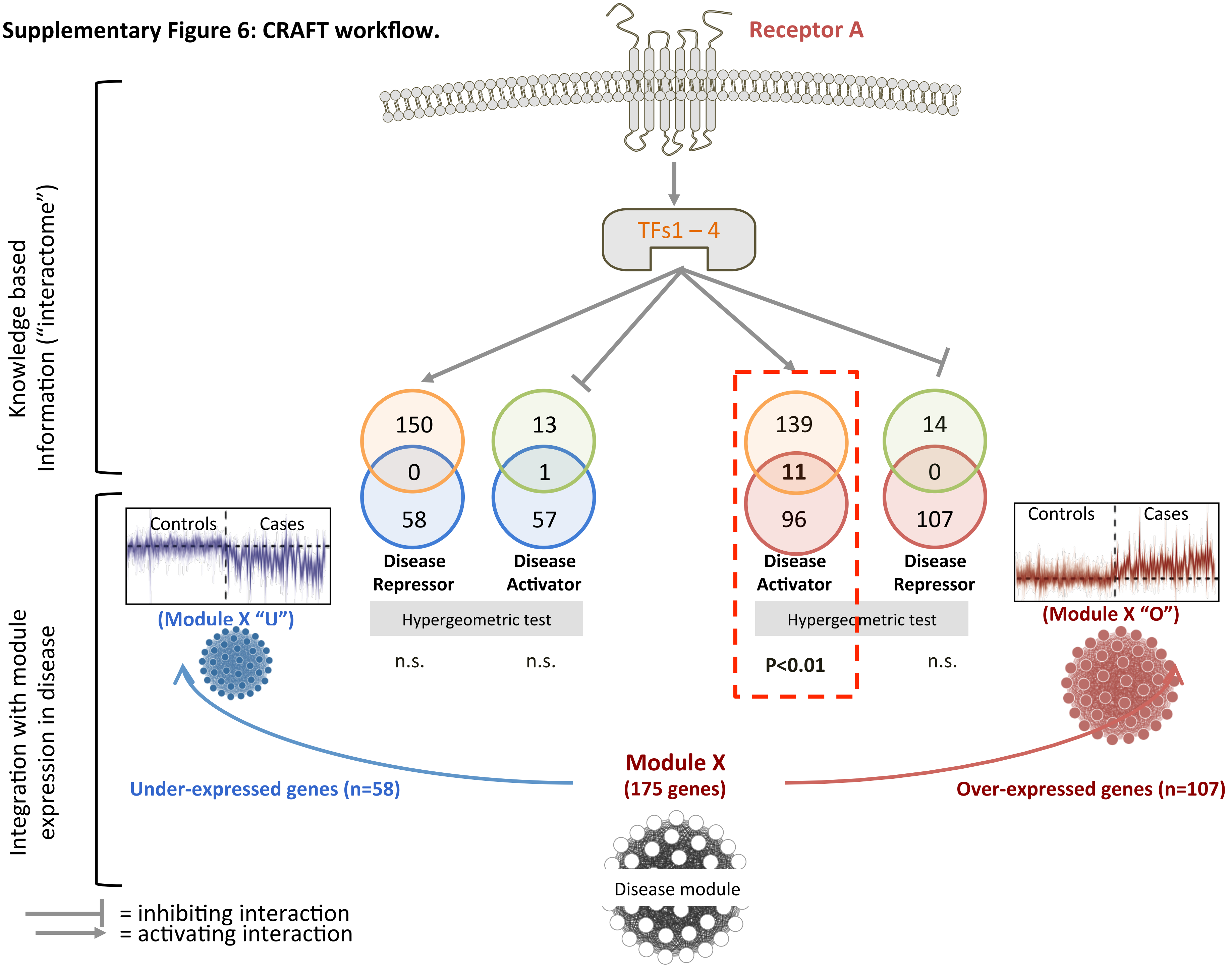

Supplementary Figure 6: CRAFT workflow. In this example we consider the effect of a single receptor "Receptor A" on the expression of genes in "Module X". Module X consists of 175 genes of which 107 are over- ("O") and 58 are under- ("U") expressed in epilepsy. In the example we consider only the set of transcription factors that Receptor A activates, corresponding to the top half of the diagram in **Figure 3**. Receptor A activates 4 TFs (labelled TFs1-4). These 4 TFs in turn influence the expression of 164 genes genome-wide, of which the expression of 150 are "activated" by one or more TF (from the list of receptor target TFs1-4) and the expression of 14 genes are "inhibited" by one or more of the TFs. The significance (and directionality) of the predicted influence of Receptor A on module gene expression is quantified by considering the overlap between direction-specified receptor effects on gene expression (via TFs1-4) with the genes in the module that are either over- or under-expressed in disease (hypergeometric test). In the example provided, 11 of the 107 genes in Module X that are over-expressed in disease overlap with the 150 genes activated by Receptor A. The significance of this overlap is calculated using a hypergeometric test and so here, Receptor A is predicted to have a significant ($P<0.01$) positive transcriptional influence on the portion of the genes in module X that are over-expressed in disease (note that the set of genes in a module which are over- or under-expressed in disease are termed "sub-modules"). Since these genes are over-expressed in disease compared to control, further increased expression of these genes by Receptor A is anticipated to make the disease worse, and so Receptor A can be designated a disease activator and therefore the therapeutic approach would be to block Receptor A.

Supplementary Figure 7a: Cell surface receptor target enrichments.

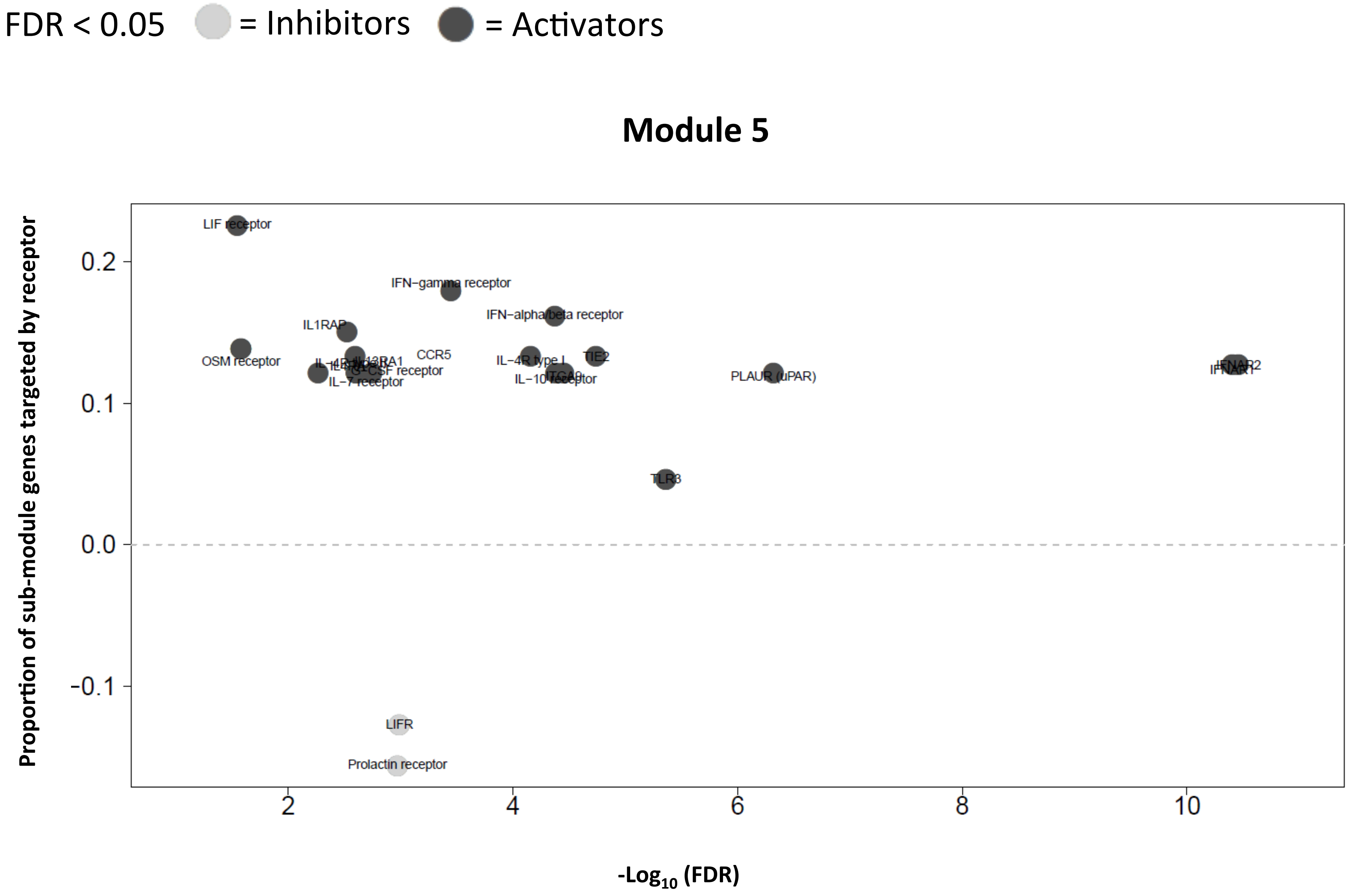

Supplementary Figure 7b: Cell surface receptor target enrichments.

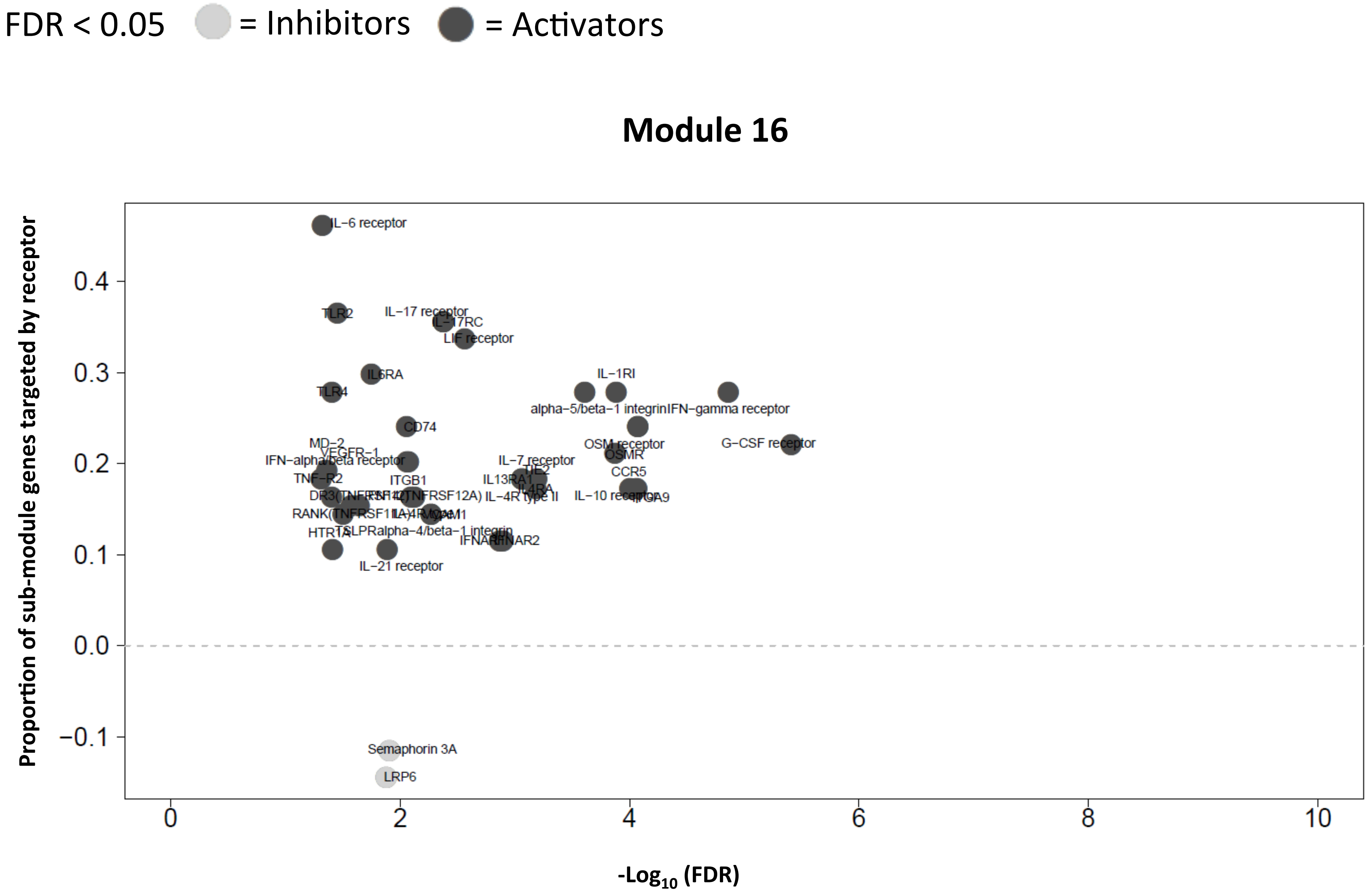

Supplementary Figure 7c: Cell surface receptor target enrichments

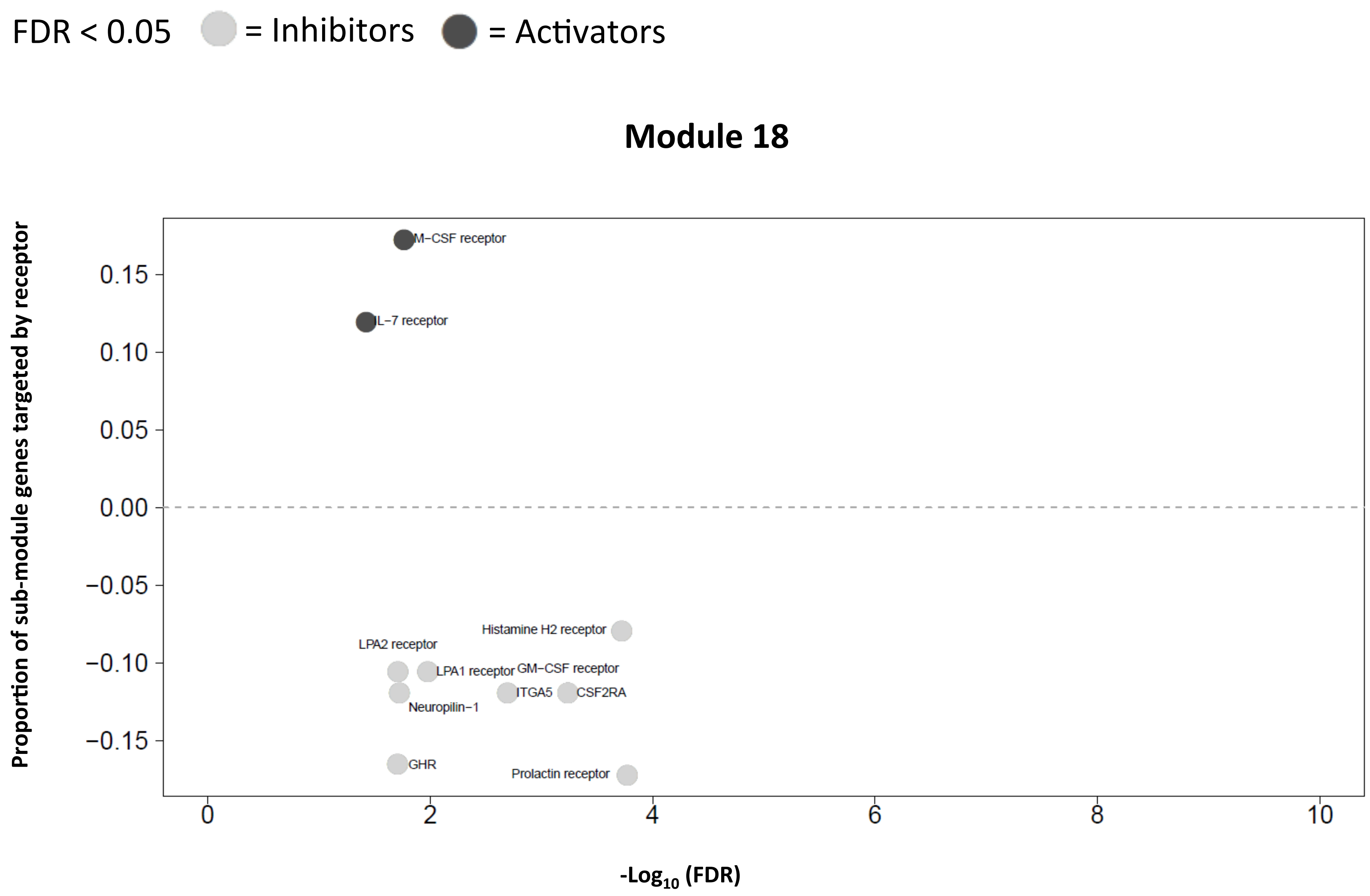

Supplementary Figure 7d: Cell surface receptor target enrichments

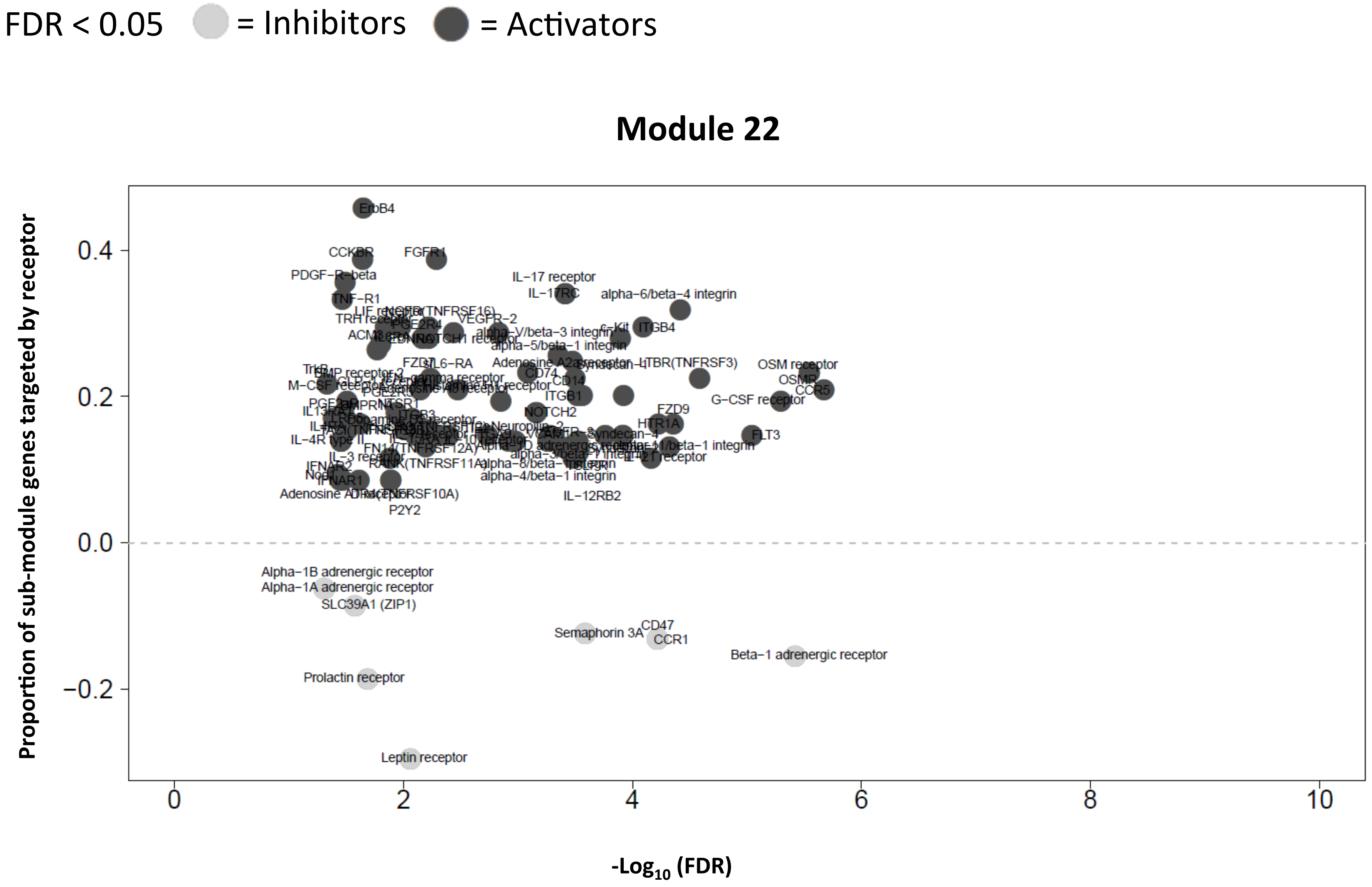

Supplementary Figures 7: Cell surface receptor target enrichments. Plot of the proportion of genes in a sub-module targeted by a (named) membrane receptor against the network's $–\log_{10}$FDR of receptor target enrichment. Receptors above the dotted line are Activators whilst those below the line are Inhibitors. Note that Activating or Inhibitor here refers to the effect of the receptor on the set of module genes which are over- or under-expressed in epilepsy (i.e., "sub-modules") and not the effect of the receptor on epilepsy, which is itself inferred according to the Causal Reasoning Rules detailed in **Figure 3**.

Supplementary Figure 8: Iba1+ immunolabeling.

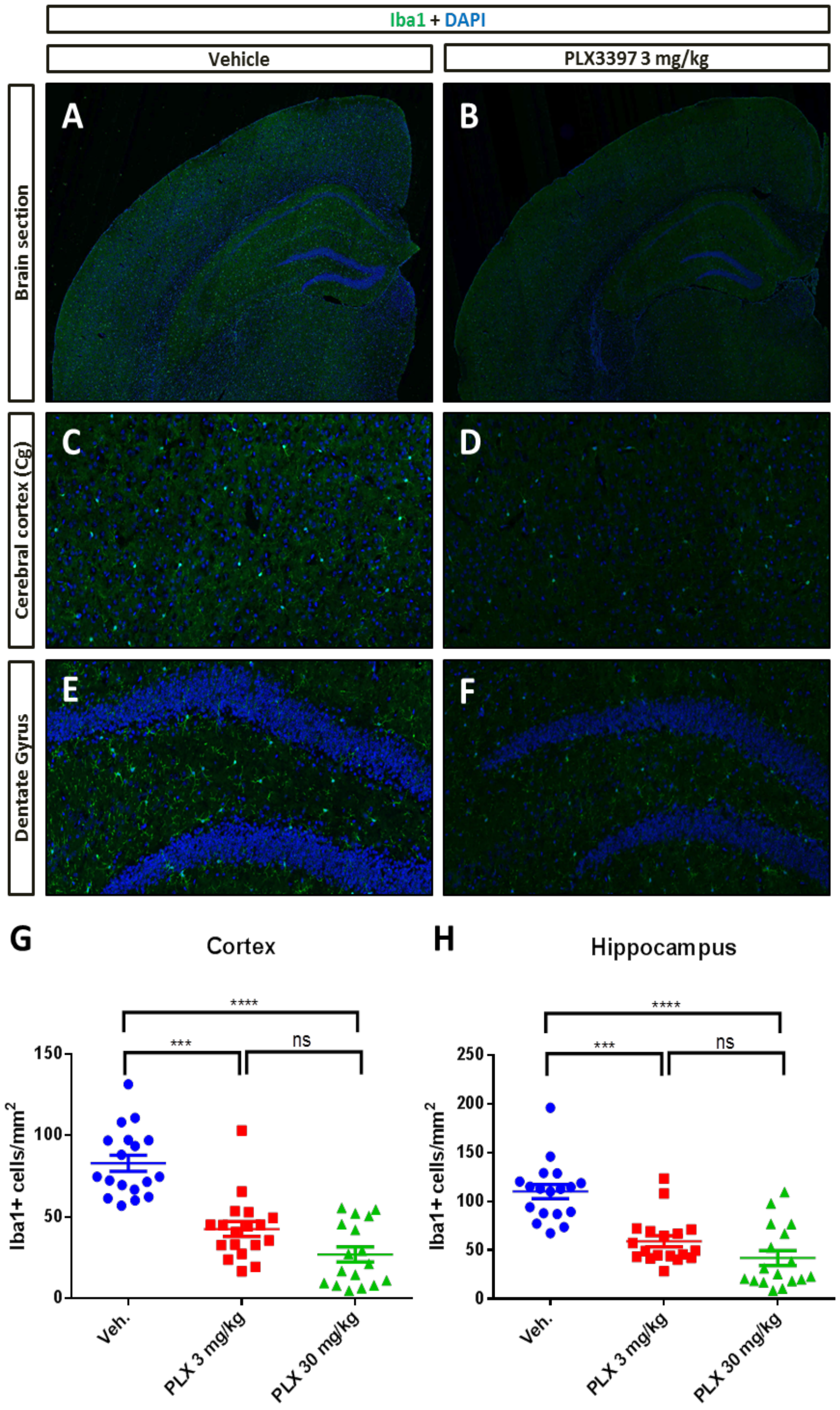

Supplementary Figure 8: Iba1+ immunolabeling. Epileptic mice were treated with PLX3397 (3 or 30 mg/kg/day) or vehicle for 7 days (n=17-18 animals per group). Epileptic mice treated with PLX3397 show a robust decrease in Iba1 immunolabeling in the brain (Panels A, B). There is a strong decrease in Iba1+ cell number in the cerebral cortex (C,D) and dentate gyrus (E,F). Quantification of the number of Iba1+ cell bodies, expressed as cell density, from 3 sections in the hippocampus (G) and cerebral cortex (H), using 3 mg/kg and 30 mg/kg PLX3397. Statistical analysis was performed using Kruskal-Wallis test followed by Dunn's test for comparison of each group to control (vehicle). Bars represent mean ± SEM. *** $p<0.001$; **** $p<0.0001$. Cg: cingulate gyrus. Ns: not significant, $p>0.05$. Scale bar in A,B = 500 µm; scale bar in C,D,E,F = 100 µm.

Supplementary Figure 9: Module enrichments for genes significantly (FDR<5%) down regulated by PLX3397 in epileptic mice.

A) **Down-regulated genes (30mg)**

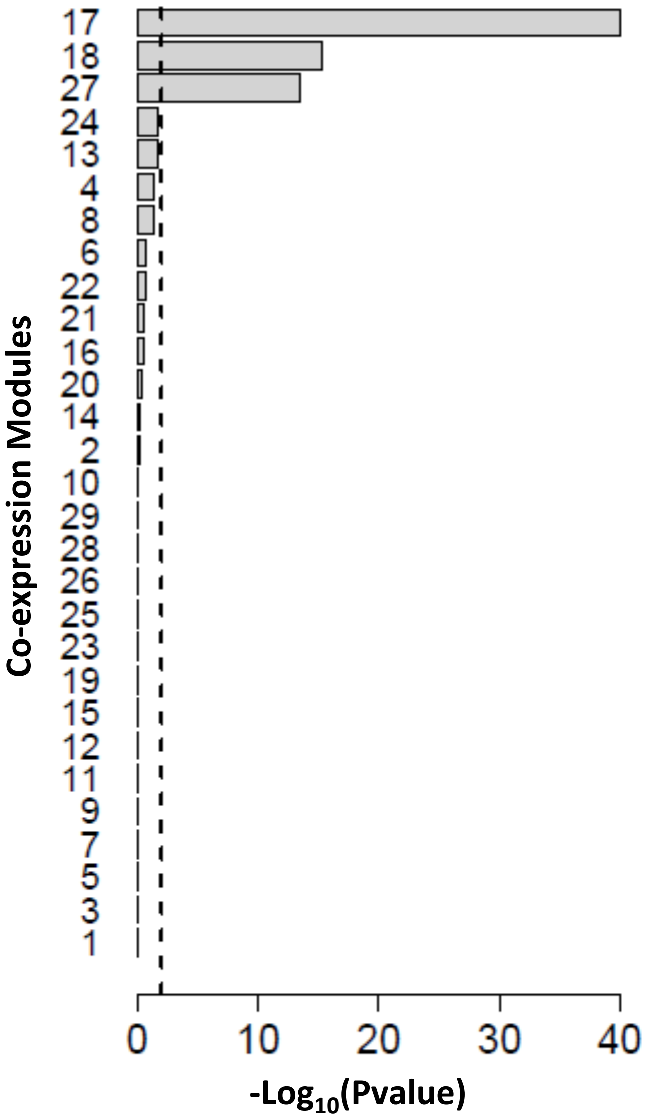

B) **Down-regulated genes (3mg)**

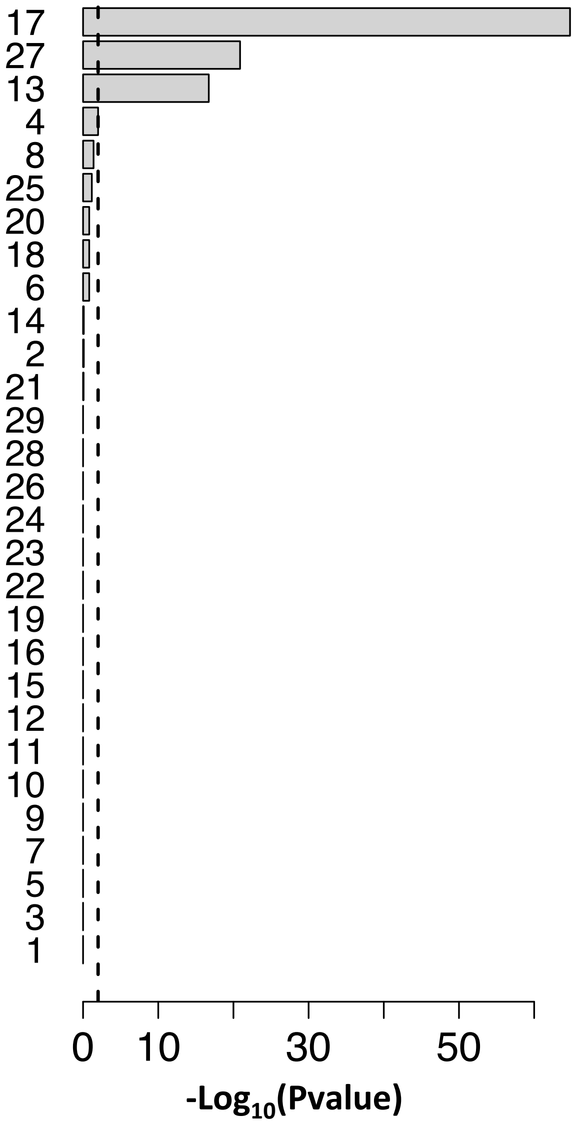

Supplementary Figure 9: Module enrichments for genes significantly (FDR<5%) down regulated by PLX3397 in epileptic mice. Figure summarizes the module enrichments for genes down-regulated by PLX3397 at 30mg/kg/day (left) and 3mg/kg/day (right). The dotted black line represents FDR<0.01.

Supplementary Figure 10:
Assessment of microglial cell death.

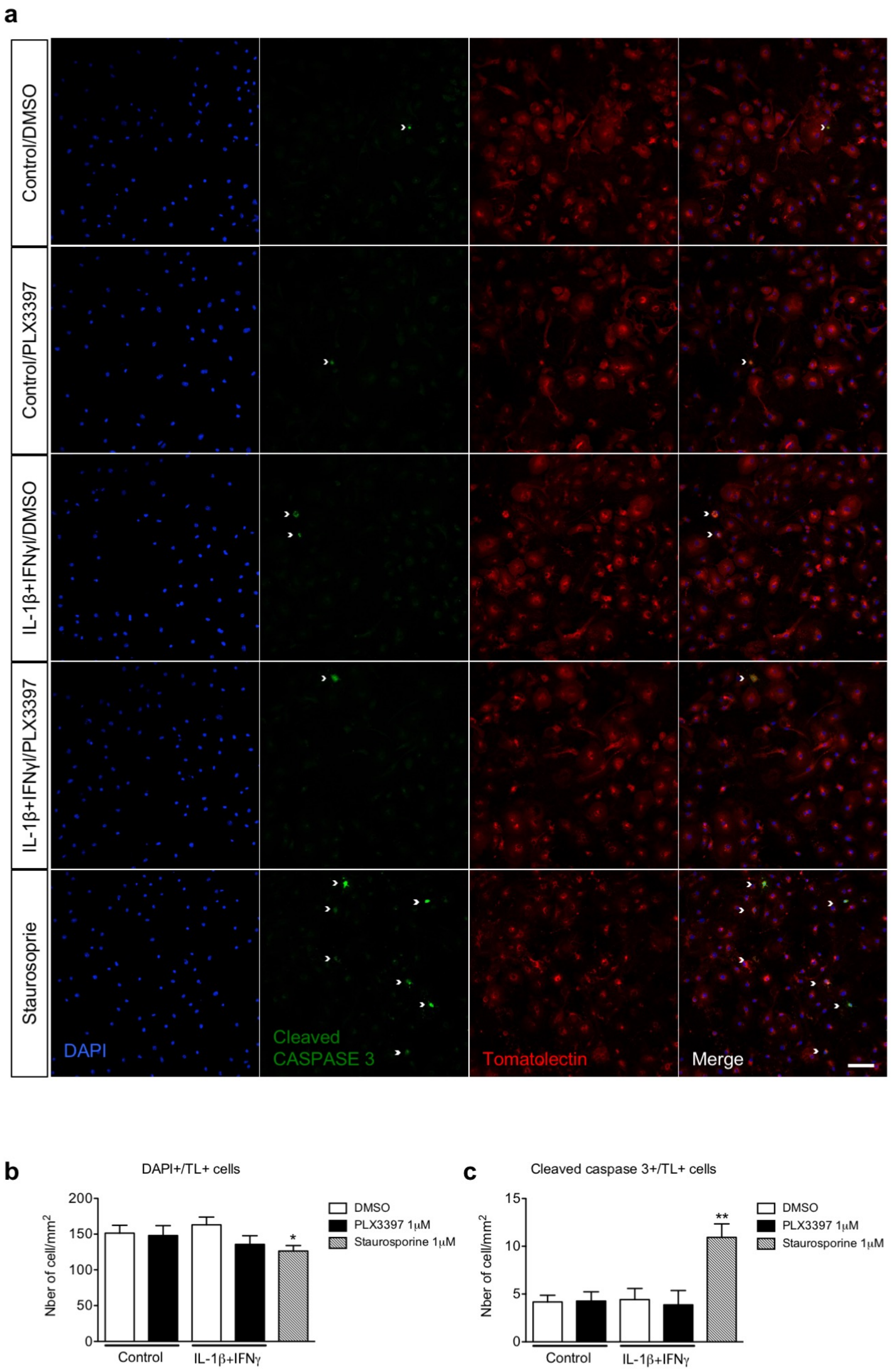

Supplementary Figure 10: Assessment of microglial cell death. (A) Representative images of DAPI, Tomatolectin (TL, for selective labeling of microglial cells) and cleaved caspase 3 staining in primary mouse microglia in all conditions. Scale bar: 70μm. **(B)** Quantification of the number of the DAPI nucleus/mm^2. **(C)** Quantification of the number of the cleaved caspase 3/mm^2. All cells were TL+ microglia. Staurosprorine is an inducer of apoptosis and was used as positive control and induced a significant reduction of the number of microglia (b) and increased the expression of cleaved caspase 3 (c) (*p<0.05, ** p<0.01 Mann Whitney test as compare to DMSO, n=4-5/ group).

Supplementary Figure 11: PLX3397 pharmacokinetics.

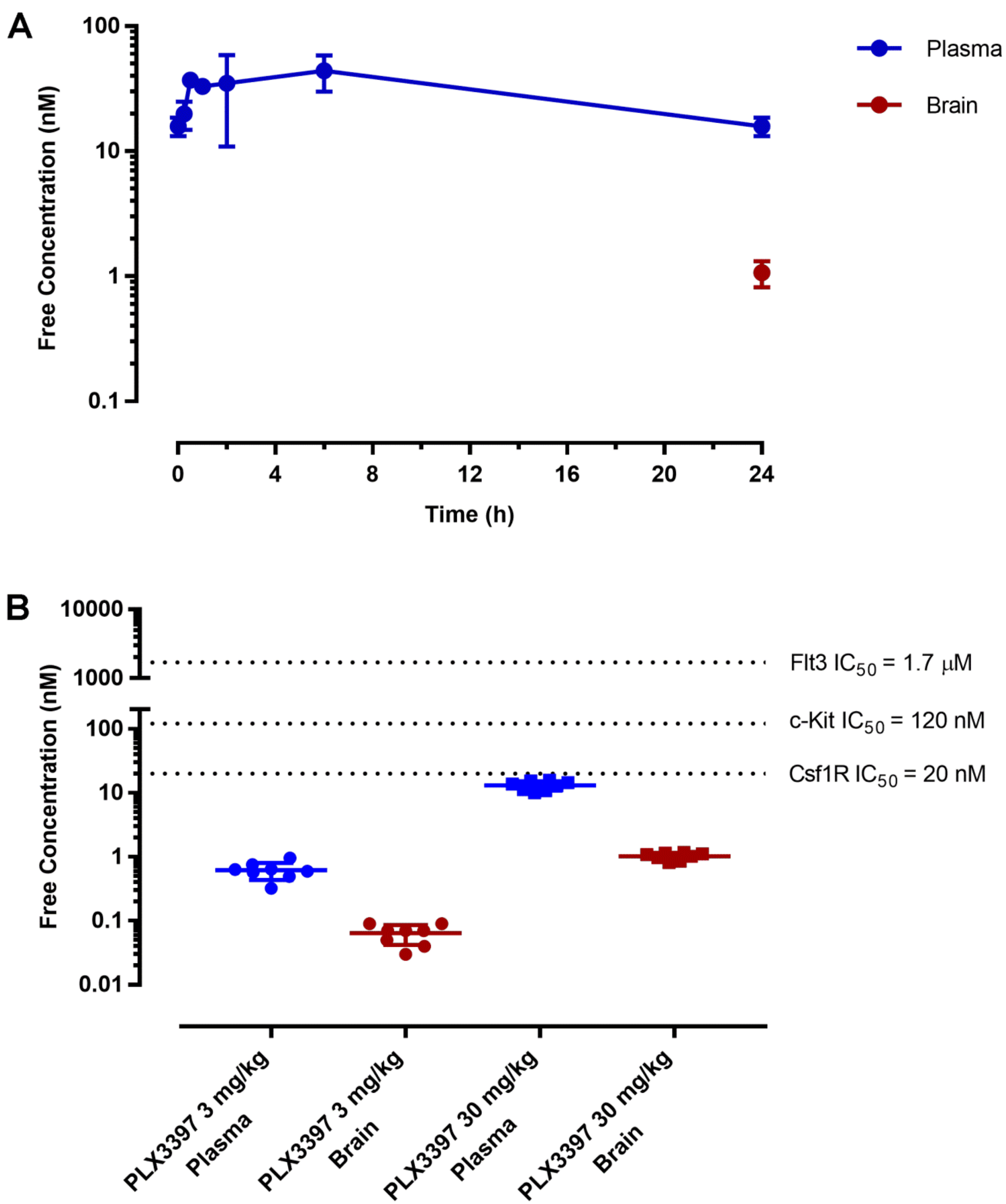

Supplementary Figure 11: PLX3397 pharmacokinetics. (A) Free (unbound) concentration in mouse plasma and brain following PLX3397 treatment at the therapeutic dose of 30 mg/kg/day. Free plasma concentrations are between 15-44nM in the 24-hour period between doses. Mice were then sacrificed after 24 hours of monitoring and free brain concentration was found to be about 1nM. **(B)** Terminal plasma and brain concentration in sample collected at steady state after 7 days of dosing (3 and 30 mg/kg/day oral gavage) in epileptic mice (pilocarpine model). Mean free plasma concentrations measured 24 hours after the last administration were 0.6 ± 0.2 nM and 13 ± 2 nM for 3 and 30 mg/kg doses respectively, and mean free brain concentrations were 0.06 ± 0.02 nM and 1.0 ± 0.13 nM respectively. Dotted lines highlight IC_{50}s for Csf1R, c-Kit and Flt3 receptors.

Supplementary Figure 12A: PLX3397-treated (30mg/kg) epileptic mice *ex vivo* microglial phagocytosis assay.

Mean ± s.e.m.
Unpaired t-test
*P < 0.05
n = 20-24 slices/group
covering 3 animals/group

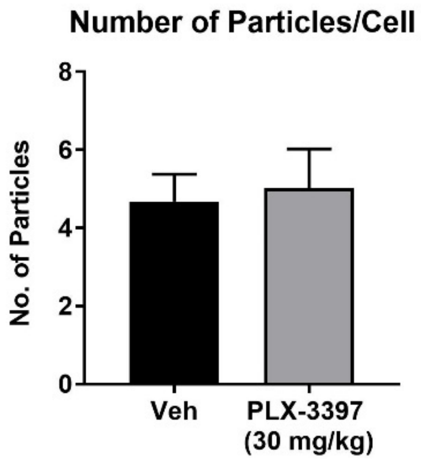

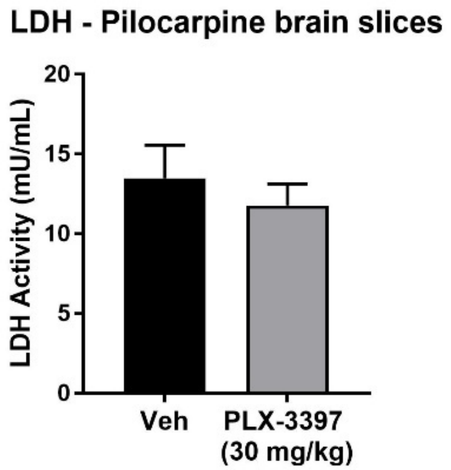

Supplementary Figure 12B: Morphology analysis of microglia in vehicle- and PLX3397-treated epileptic mouse brain.

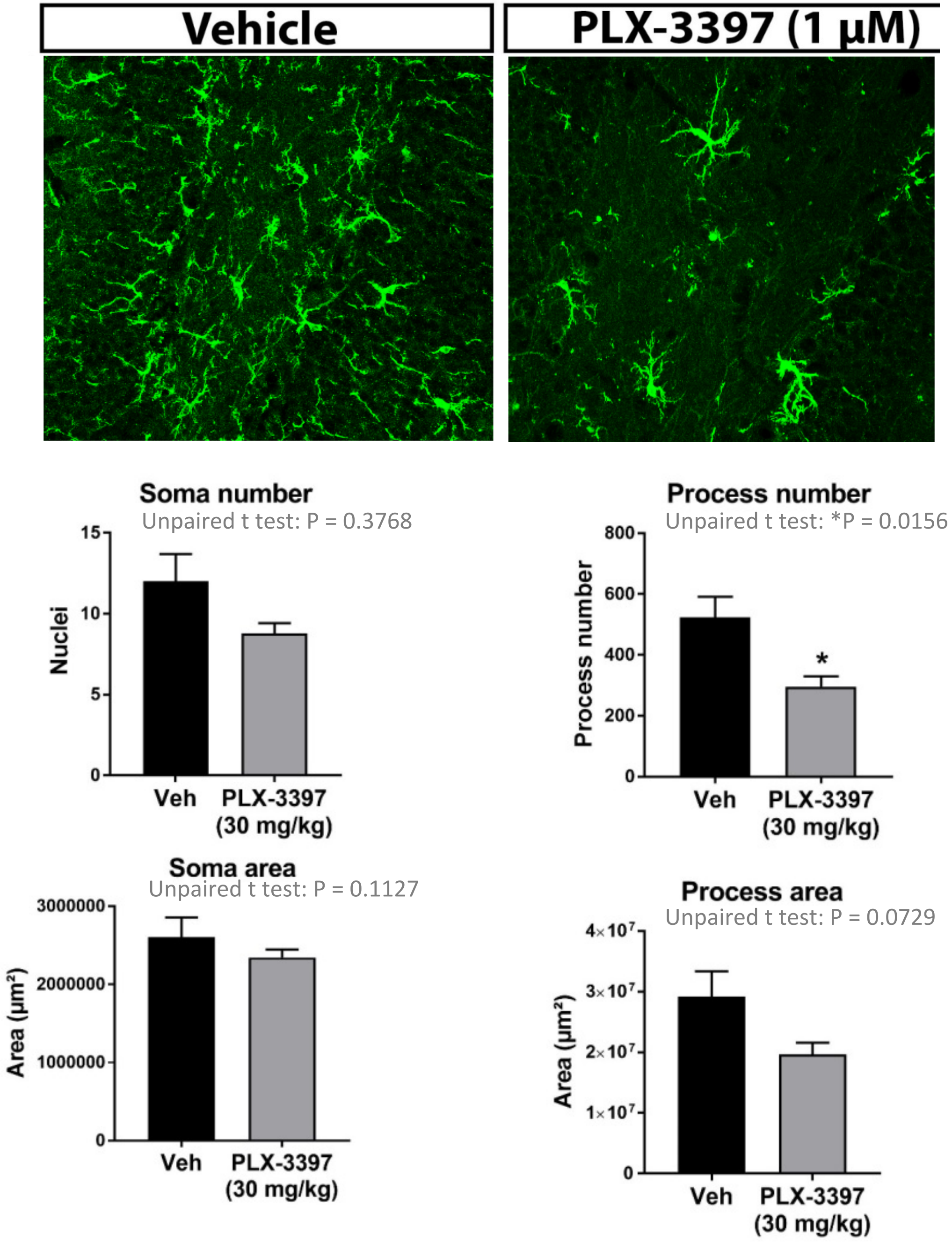

Supplementary Figure 12C: PLX3397 (1 μM) reduces primary microglial cell migration.

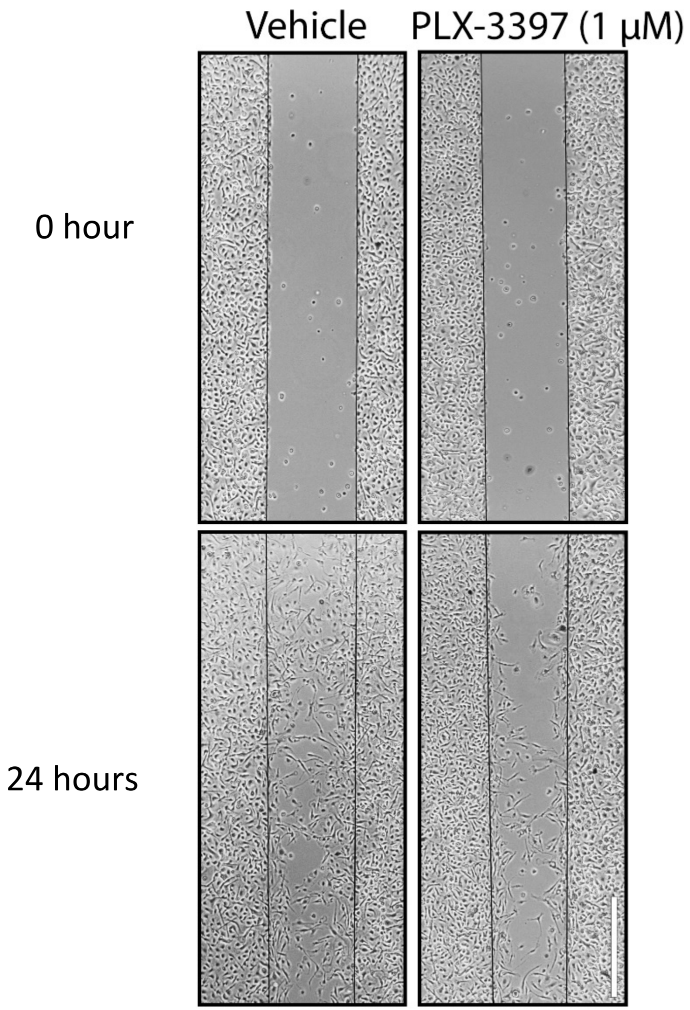

Scale bar = 500 um

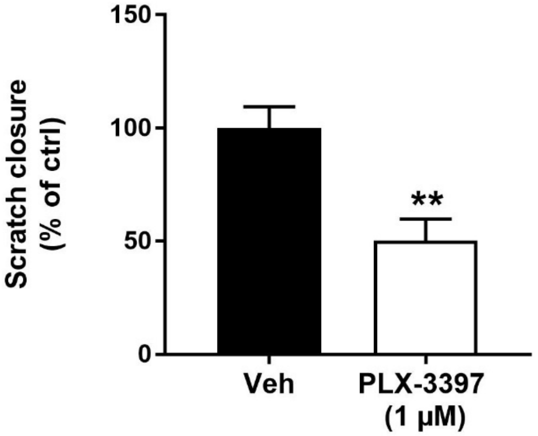

Mean ± s.e.m.
Unpaired t-test,
*P = 0.0039
n = 6 wells/treatment

Supplementary Figure 12D: *In vitro* microglia live (green) dead (red) assay.

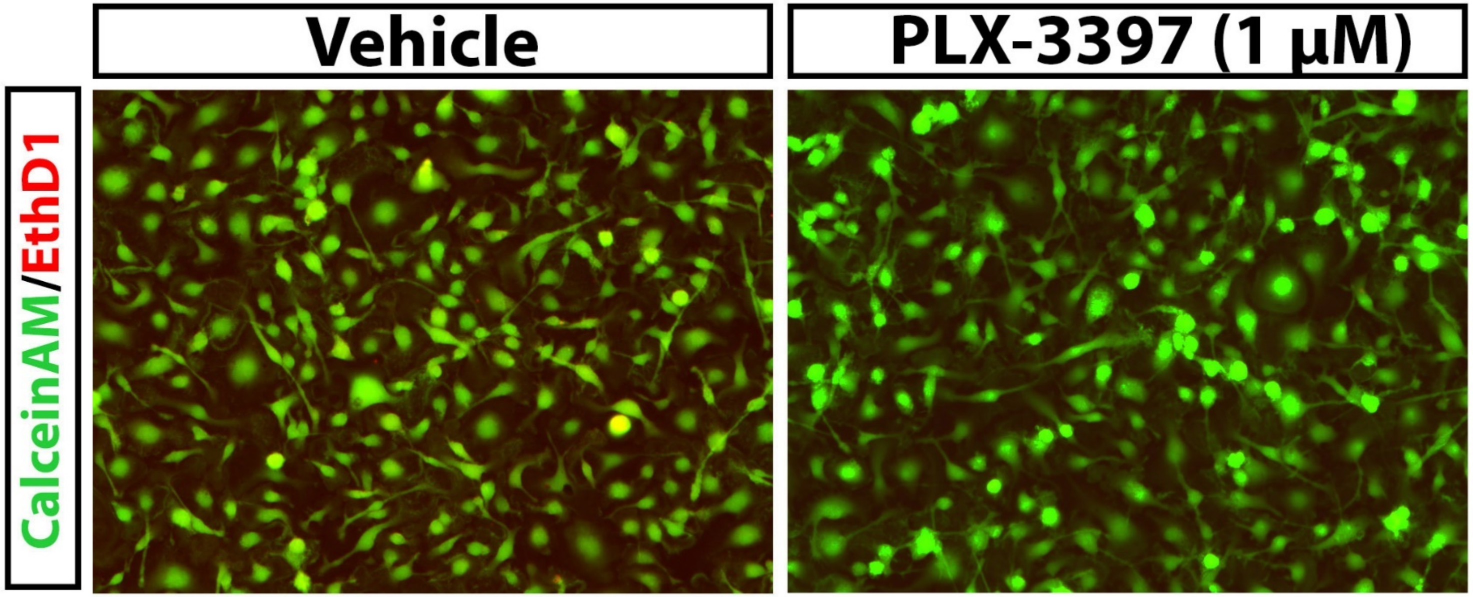

Supplementary Figure 12: (A) PLX3397-treated (30mg/kg) epileptic mice *ex vivo* microglial phagocytosis assay. Number of pH Rhodamine-conjugated zymosan beads (red) taken up per microglia cell (Iba1 staining) in vehicle and PLX3397-treated epileptic mouse brain sections revealed no differences in the number of particles taken up per cell. Levels of extracellular LDH in culture fluids of acute brain slices in vehicle and PLX3397 treated epileptic mouse brain slices revealed no evidence for PLX3397-induced microglial cell death. **(B) Morphology analysis of microglia in vehicle- and PLX3397-treated epileptic mouse brain.** Slices showing that microglia exposed to PLX3397 have thicker healthier processes compared to the highly filamentous discontinued filopodia in vehicle alone treated epileptic mice. **(C) PLX-3397 (1 µM) reduces primary microglial cell migration.** Assessment of PLX3397 on microglia migratory function using scratch assay. Cells were plated in 96 well plate format containing delineated boundaries by a plastic scaffold. Following treatment with PLX3397 the scaffold was removed and cells were allowed to migrate across the empty space previously occupied by plastic. At 24h microglia migration into the space was measured. Data from 3 independent experiments show significant decrease on motility of microglia exposed to 1µM PLX3397. **(D) *In vitro* microglia live (green) dead (red) assay.** Live (green)/dead (red) cell assay performed on the same primary microglia cells used for the scratch assays showing very low numbers of dead cells in culture (i.e., 1µM PLX3397 does not impact the viability of primary mouse microglia).

Supplementary Figure 13:
Effect of PLX3397 on seizures in acute seizure models.

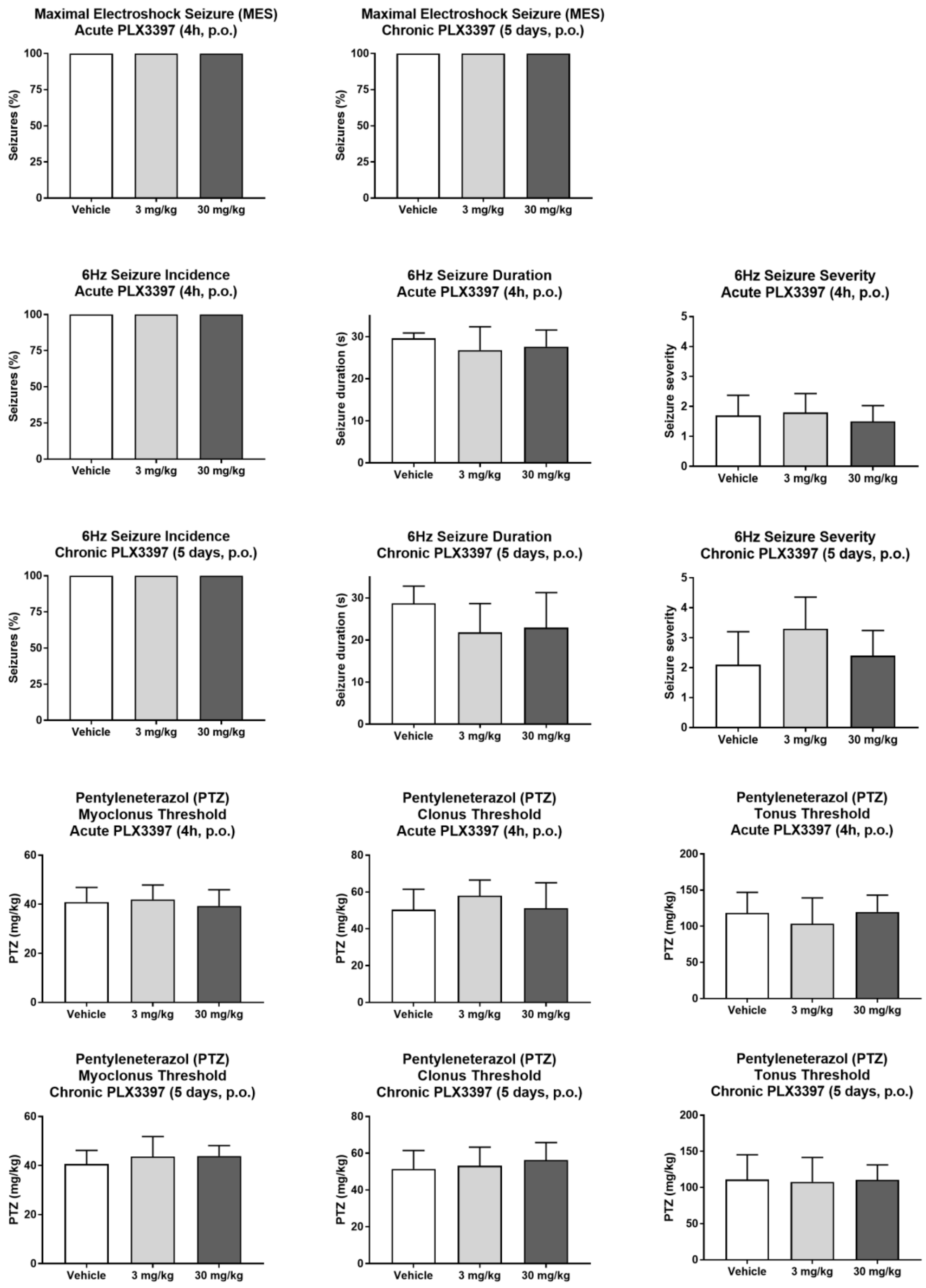

Supplementary Figure 13. Effect of PLX3397 on seizures in acute seizure models. We studied the following acute seizure models: the Maximal Electroshock Seizure (MES) model, the 6Hz psychomotor model and the pentylenetetrazol (PTZ) seizure model. Prior to acute seizure induction the mice were treated with either single dose PLX3397 (30mg/kg) or received 5-days of pre-treatment with PLX3397 (30mg/kg/day) p.o. In both acute dose and chronic pre-treatment PLX3397 failed to display any anticonvulsant activity in any of the models and across a range of outcomes, supporting a disease-context specific anticonvulsant effect of PLX3397.

Supplementary Figure 14: Post-hoc power calculation for number of mice required for differential co-expression network analysis.

A) Distribution of distances between epileptic and control mice for varying sub-samples

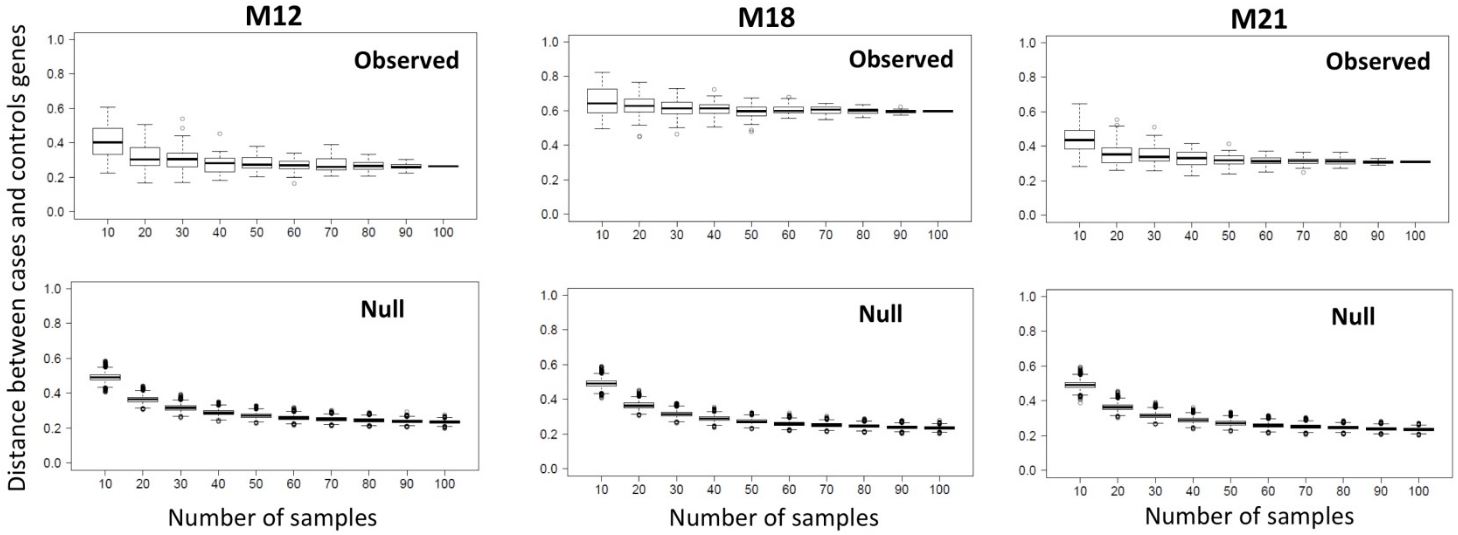

B) Empirical significance (*P*-value) to detect differential co-expression

Sample size (cases and controls)	M12 (effect size = 0.27) P-value	M18 (effect size = 0.6) P-value	M21 (effect size = 0.31) P-value
**10**	0.7792	0.0354	0.7115
**20**	0.7215	< 0.00001	0.5895
**30**	0.5902	< 0.00001	0.2892
**40**	0.5809	< 0.00001	0.2330
**50**	0.4699	< 0.00001	0.0886
**60**	0.3714	< 0.00001	0.0303
**70**	0.3740	< 0.00001	0.0153
**80**	0.2488	< 0.00001	0.0003
**90**	0.1007	< 0.00001	< 0.00001
**100**	0.0011	< 0.00001	< 0.00001

Supplementary Figure 14: Post-hoc power calculation for number of mice required for differential co-expression network analysis. (A). Distribution of distances between epileptic and control mice for varying sub-samples for three representative differential co-expression modules associated with epilepsy (M12, M18 and M21) which are each of similar size in terms of the number of genes but which vary in terms of their mean gene-gene correlation (see Methods for details). **(B)** Empirical significance (P-value) to detect differential co-expression between epileptic and control hippocampi in mice for the three representative modules.
