# Supplementary material for: A systems-level framework for drug discovery identifies Csf1R as an anti-epileptic drug target
Source: Nat Commun. 2018 Sep 3;9:3561. doi: 10.1038/s41467-018-06008-4 (PMC6120885; doi:10.1038/s41467-018-06008-4)
Supplement: Supplementary file 3 — Description of Additional Supplementary Files [file 41467_2018_6008_MOESM3_ESM.pdf]

Description of Additional Supplementary Files

File Name: Supplementary Data 1
Description: List of genes differentially expressed (DE) between epileptic case and non-epileptic control mice. Genes in grey are not-significantly differentially expressed (FDR > 0.05). Log2 fold change >0 = up-regulated in epilepsy, Log2 fold change <0 = down-regulated in epilepsy.

File Name: Supplementary Data 2
Description: List of modules and constituent genes. Module 3 is the "grey cluster" consisting of unclustered genes.

File Name: Supplementary Data 3
Description: Table summarizing gene ontology enrichment analysis of the epileptic hippocampus co-expression modules ("clusters")

File Name: Supplementary Data 4
Description: Cell type enrichment analysis (for graphical representation of results see Supplementary Figure 2b).

File Name: Supplementary Data 5
Description: Differential co-expression in mouse. Table summarizing results from the permutation test for differential co-expression between mouse epileptic and control hippocampi. Twelve modules are significantly (FDR<0.05) differentially co-expressed between epileptic and control mice hippocampi.

File Name: Supplementary Data 6
Description: Summary statistics for the 29 epileptic mouse hippocampus modules. Grey – not significant at FDR 5%. * = modules differentially co-expressed in mouse and human TLE + correlated with seizures + conserved in human TLE.

File Name: Supplementary Data 7
Description: List of genes whose expression correlates with seizure frequency using Spearman's correlation. Significance was calculated by performing 10,000 permutations.

File Name: Supplementary Data 8
Description: Significance of gene-level co-citation with epilepsy (23,092 Abstracts with at least one gene-epilepsy co-citation)

File Name: Supplementary Data 9
Description: Significance of enrichment of gene-epilepsy pairs (hypergeometric test) in each module. Modules a significant enrichment are highlighted in green.

File Name: Supplementary Data 10
Description: Receptors with significant (FDR<0.05) impact on module gene expression. impact = direction of effect on sub-module expression, which can be activating (act), inhibitory (inh) or unspecified (uns). Note that activating or inhibitory here refer to the effect of the receptor on the set of module genes which are over- or under-expressed in epilepsy

(referred to as "sub-modules" in the Web Browser and Methods) and not the effect of the receptor on epilepsy, which is inferred by considering status and impact together. status = whether genes in the sub-module overlapping with genes regulated by the receptor are (o) or under (u) -expressed in epilepsy. FDR = significance of effect of a receptor on sub-module expression based on the overlap of genes under the control of the receptor and genes in the sub-module (hypergeometric test). %tf = "activity" i.e., the proportion of genes regulated by the receptor which are in the sub-module. %module = the proportion of genes in the sub-module that are under the control of the receptor. See Methods and main Text for further detail.

File Name: Supplementary Data 11
Description: Transcription factors (TFs) with significant (FDR<0.05) impact on module gene expression. impact = direction of effect on sub-module expression, which can be activating (act), inhibitory (inh) or unspecified (uns). Note that activating or inhibitory here refer to the effect of the TF on the set of module genes which are over- or under-expressed in epilepsy (referred to as "sub-modules" in the Web Browser and Methods) and not the effect of the TF on epilepsy, which is inferred by considering status and impact together. status = whether genes in the sub-module overlapping with genes regulated by the TF are (o) or under (u) -expressed in epilepsy. FDR = significance of effect of a TF on sub-module expression based on the overlap of genes under the control of the TF and genes in the sub-module (hypergeometric test). %tf = "activity" i.e., the proportion of genes regulated by the TF which are in the sub-module. %module = the proportion of genes in the sub-module that are under the control of the TF. See Methods and main Text for further detail.

File Name: Supplementary Data 12
Description: Effect of PLX3397 on expression of genes in primary microglia - basal state

File Name: Supplementary Data 13
Description: Effect of PLX3397 on expression of genes in primary microglia - activated state

File Name: Supplementary Data 14
Description: Effect of PLX3397 on expression of apoptosis pathways in primary microglia - basal state

File Name: Supplementary Data 15
Description: Effect of PLX3397 on expression of apoptosis pathways in primary microglia - activated state

File Name: Supplementary Data 16
Description: Effect of PLX3397 on expression of module 18 in primary microglia (basal and activated states).
